# Cardiovascular and connective tissue disorder features in *FLNA*-related PVNH patients: progress towards a refined delineation of this syndrome

**DOI:** 10.1186/s13023-021-02128-1

**Published:** 2021-12-04

**Authors:** Clarisse Billon, Salma Adham, Natalia Hernandez Poblete, Anne Legrand, Michael Frank, Laurent Chiche, Stephane Zuily, Karelle Benistan, Laurent Savale, Khaoula Zaafrane-Khachnaoui, Anne-Claire Brehin, Laurence Bal, Tiffany Busa, Mélanie Fradin, Chloé Quelin, Bertrand Chesneau, Denis Wahl, Patricia Fergelot, Cyril Goizet, Tristan Mirault, Xavier Jeunemaitre, Juliette Albuisson, Bordeaux-cohort collaborators, Anne Dieux, Fabien Labombarda, Sylvain Rheims, Odile Boute, André Vincentelli, Annick Toutain, Sylvie Odent, Gaetan Lesca, Marie Vincent, Juliette Piard, Maud Favier, Philippe Derambure, Patrick Edery, Susanne Thummler, Marion Gérard, Fanny Morice-Picard, Valérie Layet, Cécile Laroche, Laurent Pasquier, Elisabeth Sarrazin, Thierry Billette de Villemeur, Lucie Guyant-Marechal

**Affiliations:** 1 Département de génétique, Centre national de référence pour les maladies vasculaires rares, centre de référence européen VASCERN MSA, Hôpital Européen Georges Pompidou, AP-HP, 20 rue Leblanc, 75015 Paris, France; 2 INSERM, U970 PARCC, Université de Paris, Paris, France; 3 Service de Médecine Vasculaire, Hôpital Saint Eloi, CHU Montpellier, Montpellier, France; 4 Département de génétique médicale, Centre national de référence pour les maladies rares Neurogénétiques, Hôpital Pellegrin, CHU Bordeaux, Bordeaux, France; 5 Laboratoire de maladies rares : Génétique et Metabolisme (MRGM), INSERM U1211, Université de Bordeaux, Bordeaux, France; 6 Faculté de médecine, Université de la Sorbonne, Paris, France; 7 Service de chirurgie vasculaire et endovasculaire, Centre aortique tertiaire, Hôpital universitaire Pitié-Salpêtrière, AP-HP, Paris, France; 8 Inserm UMRS 1116 DCAC, Université de Lorraine, Nancy, France; 9 Division de médecine vasculaire et centre de compétence régional pour les maladies vasculaires rares et autoimmunes systémiques, Centre Hospitalier Régional Universitaire de Nancy, Nancy, France; 10 Centre de Référence des Syndromes d’Ehlers-Danlos non Vasculaires, Hôpital Raymond Poincaré, Assistance Publique Hôpitaux de Paris, Garches, France; 11 UMR U1179 INSERM, Université Versailles Saint-Quentin, Montigny-le-Bretonneux, France; 12 Université Paris-Saclay, Le Kremlin Bicêtre, France; 13 UMR_S 999, INSERM, Groupe hospitalier Marie-Lannelongue -Saint Joseph, Université Paris-Sud, Le Plessis-Robinson, France; 14 Service de Pneumologie, Hôpital Bicêtre, APHP, Le Kremlin-Bicêtre, France; 15 Unité de génétique médicale 2, Hôpital L’Archet, Nice, France; 16 INSERM U1245 , Normandy center for Genomic and Personalized Medicine, Normandie Univ, CHU Rouen, 76000 Rouen, France; 17 Centre de référence régional Marfan et apparentés, Centre aortique, Hôpital La Timone, AP-HM, Marseille, France; 18 Département de Génétique Médicale, Hôpital La Timone, CHU de Marseille, Marseille, France; 19 Service de Génétique Clinique, Centre de Référence Maladies Rares CLAD-Ouest, ERN ITHACA, CHU Rennes, Hôpital Sud, Rennes, France; 20 Service de génétique médicale, Hôpital Purpan, CHU de Toulouse, Toulouse, France; 21 Centre de Référence du Syndrome de Marfan et des syndromes apparentés, Hôpital des Enfants, CHU de Toulouse, Toulouse, France; 22 Plateforme de Transfert en Biologie Cancérologique, Centre Georges François Leclerc - UNICANCER- Institut GIMI, Dijon, France

**Keywords:** FLNA, Connective tissue disorder, Aortic aneurysm and dissection, Cardiovascular anomalies, Ehlers–Danlos

## Abstract

**Background:**

*FLNA* Loss-of-Function (LoF) causes periventricular nodular heterotopia type 1 (PVNH1), an acknowledged cause of seizures of various types. Neurological symptoms are inconstant, and cardiovascular (CV) defects or connective tissue disorders (CTD) have regularly been associated. We aimed at refining the description of CV and CTD features in patients with *FLNA* LoF and depicting the multisystemic nature of this condition.

**Methods:**

We retrospectively evaluated *FLNA* variants and clinical presentations in *FLNA* LoF patient with at least one CV or CTD feature, from three cohorts: ten patients from the French Reference Center for Rare Vascular Diseases, 23 patients from the national reference diagnostic lab for filaminopathies-A, and 59 patients from literature review.

**Results:**

Half of patients did not present neurological symptoms. Most patients presented a syndromic association combining CV and CTD features. CV anomalies, mostly aortic aneurysm and/or dilation were present in 75% of patients. CTD features were present in 75%. Variants analysis demonstrated an enrichment of coding variants in the CH1 domain of FLNA protein.

**Conclusion:**

In *FLNA* LoF patients, the absence of seizures should not be overlooked. When considering a diagnosis of PVNH1, the assessment for CV and CTD anomalies is of major interest as they represent interlinked features. We recommend systematic study of *FLNA* within CTD genes panels, regardless of the presence of neurological symptoms.

**Supplementary Information:**

The online version contains supplementary material available at 10.1186/s13023-021-02128-1.

## Background

Pathogenic variants in the X-linked *FLNA* gene encoding Filamin-A, a widely expressed cytoskeletal protein, lead to highly variable clinical presentations including periventricular nodular heterotopia type 1 (PVNH1, OMIM #300049). PVNH1 is an acknowledged cause of seizures [1, 2] and can be associated with cardiovascular (CV) anomalies including patent ductus arteriosus (PDA) and thoracic aortic dilation involving the Valsalva sinuses or tubular aorta. The disease is X-linked dominantly inherited and essentially affects women carrying heterozygous loss-of-function (LoF) pathogenic variants. A few hemizygous men with hypomorphic pathogenic variant or somatic mosaicism have also been described [3–6]. To date, published LoF pathogenic variants are found all along the protein and their location is not predictive of the severity of the phenotype. Filamin-A is composed with an actin-binding domain consisting of an N terminal domain (Nter) and two calponin homology domains (CH1 and CH2) as well as two ROD regions (ROD1 and ROD2) composed of Immunoglobulin-like (Ig-like) repeats (1 to 15 and 16 to 23 respectively) [7, 8]. One intervening calpain-sensitive “hinge” sequence is located between these two ROD domains (Hinge1). C-terminal domain (Cter), where dimerization of filamin is mediated, is defined with the Ig-like repeat 24 and one “hinge” sequence located between Ig-like repeats 23 and 24 (Hinge 2) [8].

In 2005, Sheen et al. [9] suggested that pathogenic *FLNA* variants could lead to connective tissue disorders (CTD) through a description of two families and nine sporadic cases with periventricular nodular heterotopia (PNH) associated with joint hyperlaxity (JHL), skin hyperelasticity (SHE) and aortic aneurysm/dissection. The authors suggested calling this condition Ehlers–Danlos variant of periventricular heterotopia. Further articles reported *FLNA*-mutated patients, presenting with PNH, JHL, SHE and other clinical features suggestive of CTD, supporting Sheen’s hypothesis [1, 10]. Hence, PVNH1 previously belonged to the Ehlers–Danlos syndromes (EDSs) family as Ehlers–Danlos syndrome (EDS) with periventricular heterotopia (EDS-PNH) [9–11]. This phenotype was excluded from the 2017 EDS international classification [12], due to predominant neurological presentation in the patients and insufficient data to differentiate PVNH1 from EDS-PNH.

Patients with CV anomalies and CTD phenotypes, sometimes associated with neurological symptoms, have been regularly referred to the National Reference Center for Rare Vascular Diseases, a tertiary care center, and finally diagnosed with PVNH1. These repeated observations led us to address the question of the frequency and distribution of CV and CTD features besides neurological clinical findings in patients carrying *FLNA* LoF variants. To answer that question we used a multiple approach strategy. First we described the characteristics of patients carrying *FLNA* LoF variants in whom genetic testing was performed because of CV or CTD features. Then we performed a comparison with the largest French cohort of PVNH1 patients, from the National Reference Diagnostic lab for filaminopathies A at Bordeaux University Hospital. Finally, we conducted a rapid review of the published literature on PVNH1 patients displaying CV and CTD features.

## Results

### Cohort 1

*FLNA* pathogenic variants were identified in 10 female Index-Cases of median age 38 years [range 14–66] (Table 1). Detailed phenotypic description is available in Table 1 and Additional file 1: Data S1.

**Table 1:** Frequency of clinical neurological, cardiovascular and connective tissue disorder features in the three cohorts

	Cohort 1 (n = 10) % (n)	Cohort 2 (n = 23) % (n)	Literature patients (n = 59) % (n)
*Neurological findings*			
Age	38 [14–66]	16 [1–60]	15 [0–71]
Seizures	50% (5/10)	70% (16/23)	46% (26/56)
Mega cisterna magna	29% (2/7)	25% (5/20)	31% (14/45)
*Cardiovascular findings*			
Aortic dilation/aneurysm	90% (9/10)	67% (10/15)	51% (27/53)
Pulmonary artery dilation/PAH	38% (3/8)	20% (2/10)	39% (18/46)
Aortic valve dysfunction	44% (4/9)	50% (10/20)	20% (10/50)
Mitral valve anomalies	67% (6/9)	11% (2/18)	41% (19/46)
Tricuspid valve anomalies (Prolapse, dysplasia)	11% (1/9)	6% (1/16)	25% (11/44)
Bicuspid aortic valve	0% (0/8)	29% (5/17)	3% (1/39)
PDA	0% (0/8)	27% (3/11)	41% (21/51)
VSD/ASD	22% (2/9)	100% (2/2)	27% (13/48)
Early-onset varicose veins	33% (3/9)	36% (4/11)	100% (1/1)
Arterial tortuosity	11% (1/9)	0% (0/5)	60% (3/5)
Arterial aneurysm/dissection	11% (1/10)	18% (2/11)	60% (3/5)
*Connective tissue disorder features*			
Joints hyperlaxity (JHL)	70% (7/10)	53% (9/17)	73% (29/40)
Skin hyperelasticity (SHE)	44% (4/9)	33% (5/15)	42% (14/33)
Spontaneous or easily bruising	38% (3/8)	23% (3/13)	24% (4/17)
Cutaneous fragility (scar)	38% (3/8)	8% (1/12)	63% (10/16)
Wall hernia	11% (1/9)	20% (3/15)	53% (9/17)
Gastro-intestinal problems	13% (1/8)	17% (2/12)	63% (12/19)
Emphysema	44% (4/9)	7% (1/15)	58% (19/33)
PNO	0% (0/10)	0% (0/16)	20% (2/10)
Scoliosis	60% (6/10)	28% (5/18)	18% (3/17)
Pectus carinatum/excavatum	0% (0/10)	0% (0/16)	25% (4/16)

**Fig. 1:**
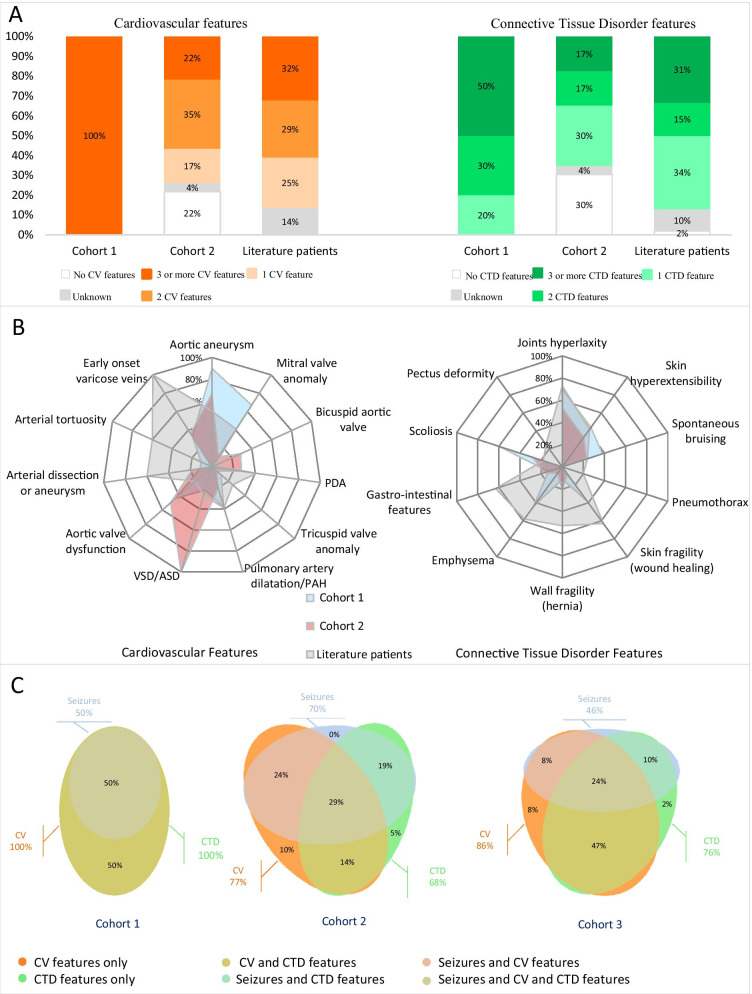
Distribution of clinical features among patients from the three cohorts. **A** Bar chart of cumulated CV or CD features in patients from the three cohorts. Most patients present two or more features of each field. **B** Radar plots summarizing cardiovascular (left panel) and Connective tissue disorder (right panel) features of the 3 cohorts. **C** Euler diagrams of clinical overlap between neurological, cardiovascular and EDS anomalies in each cohort

**Fig. 2:**
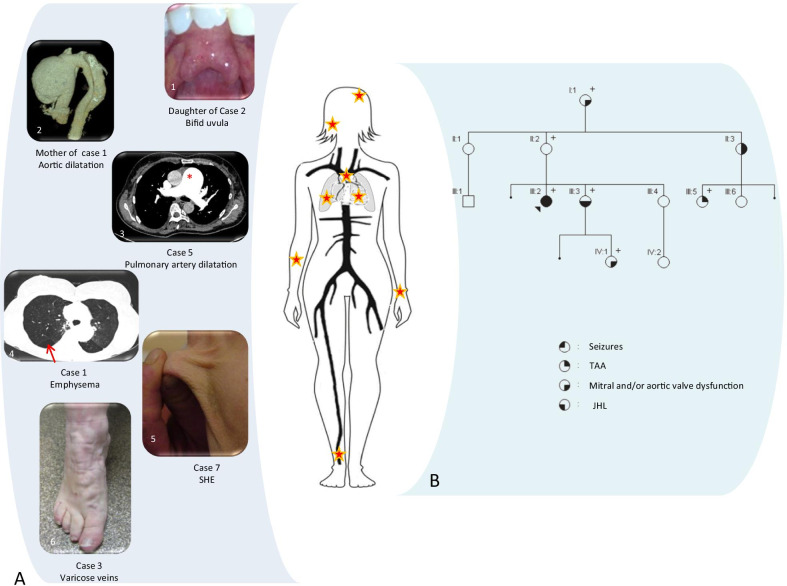
Clinical characterization of cohort 1. **A** Inter-individual phenotypic variability: Schematic representation of features in the whole body. 1/ Bifid uvula in daughter of index-case#2. Her phenotype is similar to her mothers’, including seizures, and PNH. Angio-CT scan demonstrated dilation of both the ascending aorta (43 mm) and the pulmonary artery without arterial tortuosity. 2/ Mother of index-case#1 had an unexpected 140 mm-diameter TAAA (associated to a bicuspid aortic valve not shown). She died at 62 from dissection. 3/ Pulmonary artery dilatation (55 mm) in index-case #5. She was referred for idiopathic Pulmonary arterial Hypertension (PAH) associated with syndromic aortopathy. CV examination revealed major dilatation of pulmonary artery (55 mm), aortic dilatation at the sinus of Valsalva (52 mm) and a mild mitral valvulopathy. 4/ Emphysema of unknown origin in index-case #1: She presented a TAAA, JHL, a minor scoliosis, easy bruises and enlarged scars. 5/ SHE in index-case#7: Clinical examination revealed some CTD features: JHL with major hypermobility (Beighton score 9/9), kyphoscoliosis, thin and translucent skin, spontaneous bruising and atrophic scars along with acrogeria and moderate arachnodactyly. 6/ Severe varicose veins due to chronic venous insufficiency in the foot of index-case#3. She was operated for TAAA and had pulmonary artery dilation with PAH. Clinical examination found velvety skin without SHE, JHL (Beighton score 5/9) and scoliosis along with disorders of the temporomandibular joint. **B** Intra-famillial phenotypic variability. Family 9 pedigree. Index-case #9 (III-2), 30 year-old, had an aortic aneurysm (Z-score at Valsalva Sinus = 2.44), a leaky aortic valve and JHL. Cerebral MRI previously performed for seizures had revealed PNH. Molecular analysis identified an *FLNA* pathogenic LoF variant c.6772G>T, p.(Glu2258*).Clinical evaluation of the mother (II-2) did not reveal neurological symptom, neither JHL nor SHE. CV evaluation wasn’t available. The sister (III-3) had JHL and an aortic valve insufficiency without aortic aneurysm nor seizures. The cousin (III-5) had aortic dilation (Z-score at Valsalva Sinus = 2.4), normal aortic valve function, and absence of JHL and seizures. Her mother (II-3) had a mitral valve insufficiency and an aortic dilatation (Z-score at Valsalva Sinus = 3.5) without CTD features or seizures. The grand-mother (I-1) had aortic valve dysfunction, but her complete clinical evaluation was not available. In this pedigree, none of the six relatives carrying the *FLNA* pathogenic variant had neurological symptoms

PNH was proven in all nine patients who had cerebral MRI. Only five patients presented seizures (Table 1). All patient presented CV and CTD features with a median of 3 [min–max: 3–5] CV anomalies and 3 [min–max: 1–6] CTD features (Fig. 1A, Additional file 2: Data S2). The most frequent CV anomalies were aortic dilatation/ aneurysm in nine patients, followed by mitral valve anomaly in six (Table 1, Figs. 1B and 2A). One patient had severe varicosities of the feet (Index-case #3, Fig. 2A). The two major CTD features, JHL and SHE were present in seven and four patients respectively (Fig. 1B). All patients presented a syndromic presentation with at least four clinical features belonging to CTD spectrum associated with rare vascular disorders (Fig. 1A, C). This presentation was highly variable amongst index-cases (Fig. 1A, B). Family history of these patients also demonstrated a high intrafamilial phenotypic variability.

To illustrate intrafamilial and inter-individual variability, we shortly present Index-Case#1 and pedigree #9 (Fig. 2B). Index-case # 1 was a 38-year-old female referred to investigate a syndromic aortopathy. She had a thoracic ascending aorta aneurysm (TAAA, Sinus of Vasalva Z-score = 4.13) and an aortic dissection developed on an abdominal aortic aneurysm. Several CTD features were noted after physical examination (Additional file 1: Data S1 and Fig. 2A), as well as a severe panlobular emphysema of unknown origin, not related to smoking. Family history revealed that her mother died at 62 years from a bicuspid aortic valve complicated with type A aortic dissection with a 140 mm-diameter ascending aortic aneurysm at hospital admission. Personal or family history of epilepsy was absent. This severe and syndromic presentation in two first-degree related women without risk factors led us to perform CTD genetic analysis. A motherly inherited *FLNA* LoF variant c.705G>A, p.(Trp235*) was identified. Cerebral MRI was then carried out and evidenced PNH and mega cisterna magna. Pedigree #9 is also representative of high intrafamilial phenotypic variability (Fig. 2B).

### Cohort 2

Among the 111 female Index-Cases with PVNH1, 48% (n = 53) had seizures. We selected the 23 patients for whom at least one CV or CTD feature was mentioned in the clinical database of the lab. For 79% (n = 88) the status related to CV or CTD abnormalities was unknown.

Based on HGVS and ACMG classification [13] all the variants excepted one were classified pathogenic or likely pathogenic. Complementary functional analyses for one variant (patient #2) was performed: *FLNA* cDNA sequencing on blood cells demonstrating that c.2625G>A produced a 34 bp deletion shifting the reading frame, (p.Lys876SerfsTer11) and led us to classify this synonymous variant as pathogenic (Additional file 5: Method S1). The synonymous variant in patient #20 was classified as VUS (c.5184C>T p.(Gly1728Gly)), RNA analysis was not performed.

The median age of these 23 patients was 16 years [min–max: 0–60] and their detailed phenotypic description is presented in Table 1 and Additional file 3: Data S3. Most patients were within infancy when clinical description was provided.

All 23 patients had PNH demonstrated before molecular diagnosis and 70% of patients (n = 16) had seizures. There was a median of 2 CV anomalies [min–max: 0–7] and 1 CTD feature [min–max: 0–3] in these patients. In total, 77% (n = 17/22) of patients presented CV anomalies (Fig. 1A, B, C).The most prominent CV features were aortic aneurysm in 67% of patients (10/15) with aortic insufficiency in 50% of patients (10/20), followed by bicuspid aortic valve in 29% (5/17). We did not have the information about CV features in one patient.

CTD features were described in 68% (15/22) of patients (Fig. 1C, Additional file 2: Data S2). The most prevalent features were the two major criteria for CTD, JHL in 53% (9/17) and SHE in 33% (5/15). We did not have the information for any CTD features in one patient.

Clinical presentation was highly variable (Fig. 1A, B) with the combination of seizures and CV anomalies and CTD features in 29% (6/21) of patients, seizures and CV anomalies in 24% (5/21) and CV anomalies and CTD features without seizures in 43% (9/21) (Fig. 1C).

### Literature patients

**Fig. 3:**
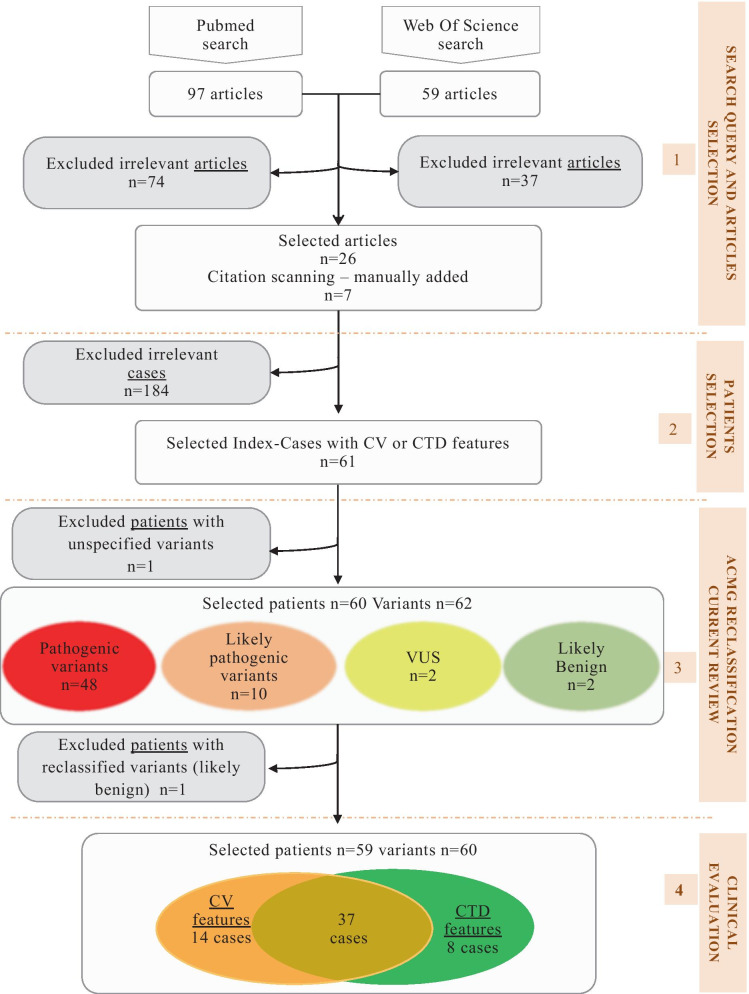
Flowchart of the article’s selection process for the literature review

We performed a rapid literature review following a multi-step process in order to build a third cohort with at least one CTD and/or CV feature from the literature (Fig. 3, Additional file 5: Method S2, Additional file 4: Data S4). After step 1 and 2 (Additional file 5: Method S2) we identified 61 cases (25%) as having unambiguous CV and/or CTD features among 245 index-cases [1–6, 9–11, 14–36].

In step 3 of our literature review, all *FLNA* variants were reclassified using HGVS and ACMG classification [13] (Fig. 3, Additional file 4: Data S4, Additional file 5: Method S2). We excluded patient #55 with unspecified variant [16]. A total of 62 variants in 60 patients were identified. After applying ACMG criteria, 48 variants were classified Pathogenic (Class 5), 10 Likely Pathogenic (Class 4). Two variants were reclassified of Unknown Significance (Class 3) in the absence of the author’s argument to classify the variant. We excluded patient #33 for which this two variants were reclassified to Likely Benign [15].

Detailed phenotypic description of these 59 index-cases is available in Table 1 and Additional file 4: Data S4. In this cohort, 66% were females. The median age was 15 years [min–max: 0–71].

PNH was proven in 93% of patients (n = 55), 46% (n = 26/56) had seizures and 31% (n = 14/45) had mega cisterna magna (information on both signs was not available for three patients). One male patient (1.6%) did not have PNH but presented with CTD and CV signs. Information was unavailable about cerebral MRI for 5% (n = 3) of patients, all presenting with CV and CTD signs. Patients had a median of 2 CV anomalies [min–max: 1–8] and 1 CT feature [min–max: 0–3].

In total, 86% (n = 51) patients presented CV anomalies (Fig. 1A, C, Additional file 2: Data S2). The most frequent anomalies were aortic aneurysm in 51% (n = 27/53), followed by PDA in 41% (n = 21/51) and mitral valve anomaly in 41% (n = 19/46). Cardiovascular information was not available or incomplete for 14% (n = 8) of subjects. In this group, males were present, and when looking for gender differences we identified an excess of tricuspid valve anomalies (prolapse or dysplasia) in males (*p* < 0.0001, Additional file 5: Table S1). We did not find difference in neurological features between males and females.

CTD features were described in 76% of patients (n = 45). Regarding major CTD criteria, 73% had JHL (n = 29/40) and 42% had SHE (n = 14/33). When reported, SHE was always associated with JHL and 10% (n = 6) had none of them. One patient did not present CTD features (patient #3). Information about all CTD features was unavailable in 10% (n = 6) of patients.

According to our literature search criteria, all patients had at least one CV or CTD feature. Overall, 86% (n = 51) of the patients had at least one CV sign and 76% (n = 45) had at least one CTD feature as previously defined (Fig. 1A), and 75% (n = 35) had both types of signs. In total, 47% of patients (n = 23) had a phenotype combining CTD and CV features without neurological symptom.

### Global analysis of PVNH1 patients with CV and CTD features

In order to perform a global analysis of clinical features in all patients, we sought for possible bias or specificities for patient’s selection by comparing the prevalence of each feature between the three cohorts (Additional file 5: Table S2).

We first compared cohort 1, mainly corresponding to an enrolment via the Reference Center for Rare Vascular Diseases, to the two other groups. The age at diagnosis was significantly higher in cohort 1 (median age = 38 years, mean age = 40 years) than in the two other cohorts (median age = 15 years, mean age = 19 years, *p* < 0.001). We did not detect any difference in the prevalence of other features (Additional file 5: Table S2). We then compared cohort 2 and literature patients, considering that the selection procedure was similar in these two groups. We excluded all features that were significantly different in the subsequent global analysis.

The analysis revealed that more than half of patients did not have seizures (n = 47/89), and that aortic aneurysm was present in 58% of patients (n = 46/78). The other remarkably frequent features were PDA in 34% of patients (n = 24/70), VSD/ASD in 29% of patients (n = 17/59), JHL in 67% of patients (n = 45/67) and SHE in 40% of patients (n = 23/57).

### Variants type and distribution in PVNH1 patients with CV and CTD features

**Fig. 4:**
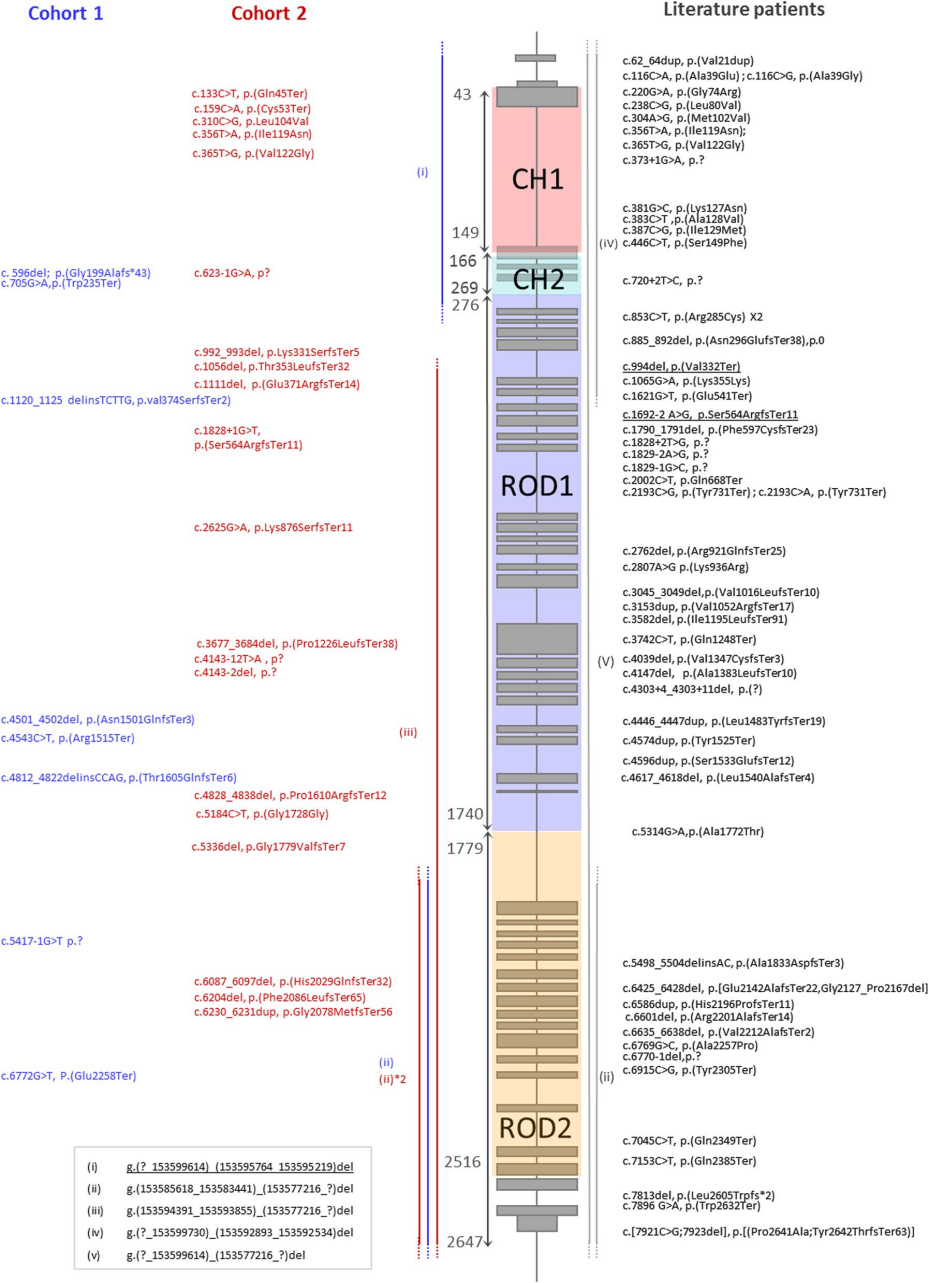
Variant type and distribution in PVNH1 patients with CV and CTD features. Schematic representation of the *FLNA* domains and overview of *FLNA* variations from PVNH1 patients with CV and CTD features (In blue: Cohort 1, in Red: Cohort 2, in grey: Literature patients). Variations with proven somatic mosaicism are underlined. Copy Number Variants are indicated with brackets, from (i) to (v) (see the bottom-left sidebar)

We gathered, analyzed, classified or reclassified a total of 93 *FLNA* variants in 92 patients with CV and/or or CTD features (Fig. 4), of which 90 could be classified as pathogenic or likely pathogenic and three as VUS (Additional file 1: Data S1, Additional file 3: Data S3 and Additional file 4: Data S4).

The most frequent variant types were frameshift (n = 34) and missense (n = 20) variants, of which one was a VUS (Additional file 5: Table S3). Large deletions, intronic, synonymous, and in-frame indel variants were rare. Amongst patients for which inheritance was known, 43% (n = 21/49) were proven de novo, and two were germline mosaicism. Information on inheritance was not available for 43 patients.

Variant distribution was globally homogeneous throughout the gene (Fig. 4). Considering that only missense variants did not eventually lead to nonsense-mediated mRNA decay and could have an impact on the protein structure or function, we studied the distribution of the 19 non-VUS missense variants within the different domains of *FLNA* regarding their relative size: N-terminal, CH1, CH2, ROD1, Hinge 1, ROD2 and C-terminal. Two domains showed significant enrichment in variant distribution regarding their relative size: the CH1 domain was highly enriched with almost two thirds of variants (n = 12, *p* = 2.289E−8) and the ROD1 domain with 5% of variants (n = 1, *p* = 0.0156). The other domains (N-terminal, CH2, Hinge 1, ROD2 and C-terminal) were not significantly enriched in pathogenic missense variants (Additional file 5: Table S4).

## Discussion

We performed a systematic analysis of some neurological and non-neurological features in patients suffering from PVNH1 caused by a *FLNA* loss of function variant. Our aim was to focus on CV and CTD features, and to delineate as precisely as possible their spectrum in PVNH1, independently of clinical or radiological PNH.

### Frequency of neurological features, age at diagnosis, and diagnostic delay

On the whole, we have observed that half of PVNH1 patients with CV and/or CTD features do not have any neurological symptom: although all but one patients included in this study presented PNH, only 50% (n = 5/10), and 46% (n = 26/56) of patients from cohort 1 and literature had seizures, respectively. In this latter group, this point should be taken with caution as patients without seizure were younger (mean 10.73 years, min–max: 0–71) than the reported mean age of onset of seizures (mean 15.75 years [1]). In contrast, the prevalence of this sign in cohort 2 (70%, n = 16/23) was highly consistent with published data [1]. Our study reinforces the knowledge of variability of neurological symptoms in this condition, and points to the associated risk of misdiagnosis and diagnostic delay. The description of patient #9 pedigree in cohort 1 is highly illustrative of this inconsistency: among six affected cases in this family only one presented with seizures, the index case who allowed definitive diagnosis in her relatives. Consistent with this finding, when evaluating age at diagnosis, we brought to light important differences among cohorts: median age at diagnosis in cohort 1 was 38 years old, compared to 16 and 15 years old respectively for the two other groups. We believe that this difference is the consequence of long diagnostic delay in cohort 1 patients, for various reasons, including the low prevalence of neurological symptoms in these patients.

### Diversity of CV and CTD features, and various syndromic presentations

We have shown that clinical presentations are highly variable among patients as well as among families, with a wide range of possible combinations in each patient (Figs. 1, 2). This observation is in line with other CTD and rare vascular disorders, where highly heterogeneous and inconsistent presentations amongst individuals are the rule.

The five most prominent features remained TAAA and JHL in more than half of patients, PDA and SHE in close to one third of patients, and VSD/ASD. A previous study focusing on CV features in PVNH1 patients showed that more than 40% of these individuals had CV features [14], mainly ascending aortic aneurysm (18%) and PDA (23%). These findings are partially consistent with our data as well as with other publications [9, 10]. However, these frequencies have limited comparability because of important differences in patient selection like inclusion of patients with documented *FLNA*-related PNH and absence of *FLNA* variants reclassification, potentially introducing a bias in the interpretation of clinical data.

Emphysema was recently identified [17, 29] as a complication of PVNH1. In our study, we specifically investigated this feature because severe emphysema in patient #1 from cohort 1 was particularly remarkable. On the whole, emphysema was found in 24 out of 57 patients included in our study, including 4 out of 10 patients in cohort 1, and more than half in literature patients (Table 1 and Additional file 5: Table S2). Recent retrospective study in a tertiary children hospital identified 6 in 9 *FLNA* LoF patients with various respiratory or lung imaging anomalies, including emphysema [37]. These observations led us to consider that this complication is still underestimated in *FLNA* LoF patients, and should be systematically investigated.

### *FLNA* molecular diagnosis

We provided a systematic analysis and reclassification of *FLNA* genetic variants in this study, and showed that the FLNA CH1 domain is significantly enriched in missense pathogenic variants. This domain was already known to be enriched in any type of pathogenic variant beyond missense variants in PVNH1 patients, whatever the clinical presentation [1]. Our analysis involved only missense variants, and confirms that the CH1 domain and its pathogenic variants are critical for FLNA function. We also show that the CH1 domain is not uniquely associated with the onset of neurological features, but in various aspects of PVNH1, including CV and CTD features. However, in light of the absence of missense variants in cohort 1, we cannot exclude that LoF variants other than missense would be associated with severe cardiovascular events. Further observations in a larger group of patients would help refining this hypothesis.

This study demonstrates a high level of de novo variants, which is another clue for a possible diagnostic delay. We identified as much as 43% patients with proven de novo variants, which imply that family history is frequently uninformative. Altogether, these facts illustrate the need to shift from a neurological-centered diagnostic process for patients harboring an *FLNA* LoF variant to a more interdisciplinary approach in order to identify these patients accurately and earlier. It could be relevant to consider this disorder as an “*FLNA* deficiency syndrome”, in place of PVNH1 which does not reflect the entirety and complexity of this condition. Systematically including *FLNA* in NGS-based CTD and CV diagnostic panels might prospectively confirm our results and refine the involvement of *FLNA* LoF variants in rare vascular and connective tissue disorders. These findings imply that physicians facing patients with CTD and CV features need to be aware of a possible *FLNA*-related disorder despite negative history of seizures. As a consequence, brain MRI should be systematically performed to diagnose PNH when an *FLNA* LoF variant is suspected or identified in a patient with CV or CTD features regardless of neurological symptoms.

## Limitations

First, the patients described in cohort 1 were referred to our Reference Center because they had a history of CV anomalies. We consider that this group is probably clinically different from cohort 2 and literature patients, because they were not recruited for neurological signs. However, the small size of this cohort did not allow us to confirm further this opinion.

Second, we built cohort 2 by selecting registry patients for whom we had information on CV or CTD signs. This information was unavailable for 88 among 111 patients, leading to a possible underestimation of the prevalence of CV complications and EDS features.

Third, Cohort 3 is also affected by possible bias. Our literature review did not follow the criteria of a systematic review. Although we followed a strict protocol for studies inclusion, exclusion, and data extraction, we did not perform any quality assessment of the selected studies. Another point relates to the criteria for article selection of PVNH1 patients with at least one CV or EDS feature. It is noticeable that we excluded 108 patients [2, 14] in the literature review because individual clinical description was not available. Moreover, the majority of patients reported in the literature may not have benefited of expert cutaneous, articular and arterial assessment, possibly underestimating the prevalence of CV complications and EDS features. The prevalence of these features in patients recruited for neurological signs can be only roughly estimated with our approach. A precise evaluation in a retrospective context would need clinical reexamination of all carriers of a Lof *FLNA* variant.

## Conclusion

Our study confirms that associated and entangled CV anomalies and EDS features are frequent and are a critical component of the clinical diagnosis of PVNH1.The presence of CTD and/or CV features without neurological symptoms is not uncommon and should not be overlooked in considering a diagnosis of PVNH1. Owing to the prevalence and severity of aortic disease in this condition, CV surveillance should be performed systematically in PVNH1 patients.

The association of JHL with or without SHE and cardiac valve anomalies and/or thoracic aorta dissection/aneurysm in the absence of seizures must trigger *FLNA* molecular analysis similarly to other CTD genes. A brain MRI can be performed for searching PVNH to support the molecular diagnosis. Despite X-linked dominant inheritance, our data suggest that evocative phenotype in male patients should not exclude the search for a *FLNA* variant.

## Patients and methods

### Cohort 1

#### Patients

The patients herein described were recruited because of CV anomalies and CTD features from 2014 to 2020 during outpatient visits in the French Reference Center for Rare Vascular diseases (Coordinating Center: HEGP, Paris, France; Collaborating Centers: Departments of Vascular Medicine of Nancy University Hospital; Internal Medicine of Clermont-Ferrand University Hospital; Pneumology of Kremlin-Bicêtre University Hospital; and Medical Genetics of Marseille, Nice, Rennes and Toulouse University Hospitals). Owing to the specificities of patients recruitment in our Centre, we did not recruit any patient because of neurological signs.

Medical information including family history and clinical findings was collected using standardized questionnaire (Additional file 5: Method S3). A total of 32 physical signs or anomalies were gathered, including neurological, CV, skin, articular and gastrointestinal features frequently found in CTD and rare vascular diseases. Physical examination was performed by a panel of clinicians specialized in CV diseases and CTD and reviewed by expert clinicians from the Coordinating Center. Clinical diagnostic criteria were ascertained as per the 2017 EDS international classification [12]. Thoracic aorta aneurysm was defined as a Z-score above 2 as described by Campens [38]. Brain MRI preceded the molecular diagnosis in four patients (patients 2, 4, 8 and 9) and was performed following molecular diagnosis in four patients (patient 1, 3, 5, 6). Blood samples were obtained from relatives whenever possible.

#### Genetic analysis

Molecular diagnosis was performed in the Genetics Department of The French Reference Center for Rare Vascular Diseases (Coordinating Center, HEGP, Paris, France), by a gene panel approach dedicated to CTD and aortopathies using Next Generation Sequencing (Additional file 5: Method S4). Patients with a *FLNA* variant of interest were selected for constitution of this cohort (variant of unknown significance, likely pathogenic and pathogenic variants according to HGVS/ACMG classification) [13]. All patients with *FLNA* variants were included in this cohort.

### Cohort 2

This cohort was built from the PVNH1 registry held by the National Reference Diagnostic lab for filaminopathies A at Bordeaux University Hospital. This registry includes patients with *FLNA* LoF variants, analyzed from 2005 to 2020 (n = 111 index-cases), mainly recruited through neurological symptoms, or PVNH discovered on brain MRI at any age.

Molecular analyses of these patients are detailed in Additional file 5: Method S1. We extracted from this registry patients with at least one CTD or CV anomaly whose medical records were retrospectively reviewed using the same questionnaire as in cohort 1.

### Literature patients

#### Literature review and patients’ evaluation process

A rapid review of the literature was conducted to (1) identify precisely and unambiguously PVNH1 published cases presenting with at least one CV and/or CTD feature (2) confirm the pathogenicity of *FLNA* variants in these patients, according to the most recent HGVS/ACMG criteria and (3) precisely evaluate the prevalence of each CTD and CV feature in these patients. The flowchart of this process is detailed in Fig. 3. The complete procedure of articles, patients, and variants selection is detailed in Additional file 5: Method S2.

### Delineation of the recurrent CTD and CV features in PVNH1

A first global analysis of cohorts 1, 2 and published cases led to the identification of 29 features of which 10 CTD and 12 CV anomalies were found once or more. They were completed by the three neurological features mentioned in the questionnaire (seizures, PNH and mega cisterna magna). The 7 remaining features were removed from further analyses. The by-cohort analysis was then led on this subset of 25 features.

### Statistical analysis

Data were analyzed using Excel 2016 software (Microsoft^®^) and RStudio 2017 software (RStudio, Inc.). Data are presented as percentages and numbers for qualitative variables and as median and extreme values for continuous variables. Percentages were calculated after excluding patients for which the data were unknown. To analyze patients’ characteristics between the three cohorts, time-independent qualitative variables were compared through logistic regression. Chi-squared test or Fisher’s exact test (whenever expected frequencies were < 5) were performed to compare variables according to sex. Fisher’s exact test of independence was also performed to compare the distribution of variants in specific domains, defined as the ratio of mutated residues over all residues in each domain. Age was the only quantitative variable and it was studied using Student’s test. All *p* values were two-sided and considered significant if < 0.05.

## Supplementary Information

**Additional file 1: Data S1.** Cohort 1.

**Additional file 2: Data S2.** Data for Venn Diagram.

**Additional file 3: Data S3.** Cohort 2.

**Additional file 4: Data S4.** Literature patients.

**Additional file 5: Method S1.** Detailed genetic analysis of Cohort 2 patients. **Method S2.** Detailed literature review, patients evaluation and variants classification process of Literature patients. **Method S3.** Questionnaire used to gather clinical information on CV and CTD features. **Method S4.** Detailed genetic analysis of Cohort 1 patients. **Table S1.** Neurological, cardiovascular and EDS features compared between male and female patients from the literature. **Table S2.** Statistical analysis of features among the three cohorts. **Table S3.** Type of FLNA variants in the three cohorts. **Table S4.** Distribution of the pathogenic and probably pathogenic missense variants in FLNA protein domains from the three groups of patients. **Table S5.** Table of abbreviations

## Data Availability

All data generated or analyzed during this study are included in this published article and its supplementary information files.
