# Supplementary material for: Cardiovascular and connective tissue disorder features in FLNA-related PVNH patients: progress towards a refined delineation of this syndrome
Source: Orphanet J Rare Dis. 2021 Dec 4;16:504. doi: 10.1186/s13023-021-02128-1 (PMC8642866; doi:10.1186/s13023-021-02128-1)
Supplement: Supplementary file 5 — Additional file 5: Method S1. Detailed genetic analysis of Cohort 2 patients. Method S2. Detailed literature review, patients evaluation and variants classification process of Literature patients. Method S3. Questionnaire used to gather clinical information on CV and CTD features. Method S4. Detailed genetic analysis of Cohort 1 patients. Table S1. Neurological, cardiovascular and EDS features compared between male and female patients from the literature. Table S2. Statistical analysis of features among the three cohorts. Table S3. Type of FLNA variants in the three cohorts. Table S4. Distribution of the pathogenic and probably pathogenic missense variants in FLNA protein domains from the three groups of patients. Table S5. Table of abbreviations [file 13023_2021_2128_MOESM5_ESM.pdf]

Supplemental Materials and tables

Supplemental method 1: Detailed genetic analysis of Cohort 2 patients

Mutation screening was performed either by DHPLC or HRM followed by Sanger sequencing (ABI, Life technologies) of all the exons showing an abnormal profile (Solé *et al*, 2009). For most recent studies the 47 coding exons and exon-intron boundaries of gene *FLNA* (NM_001110556.1) were co-amplified (AmpliSeq library Thermo Fisher Scientific) and new generation sequencing was performed on Ion PGM or S5 XL systems (Thermo Fisher Scientific). All variants were evaluated according to ACMG standards and guidelines (Richards *et al*, 2015) and numbered following HGVS recommendations. *In Silico* analysis was performed for missense and splicing variants using the Alamut visual interface (Sophia Genetics), HGMD® Online (QIAGEN digital Insights) and public databases GnomAD and ClinVar. RNA studies were performed from whole blood samples (PAXgene tube, PreAnalytiX) according to routine procedures for RNA extraction (PAXgene blood RNA kit, QIAGEN), RT-PCR (SuperScript®III first strand synthesis system, Stratagene) and Sanger sequencing. Gene dosage anomalies were identified by dedicated microarray comparative genomic hybridization (aCGH) and CytoGenomics software (Agilent Technologies)(26).

Supplemental method 2. Detailed literature review, patients evaluation and variants classification process of Literature patients

As a first step, Pubmed and Web of Science screening (Queried on October 2020) using Boolean operators and specific terms for CTD and CV features in PVNH1 ((FLNA OR FLN1 OR filamin-1 OR filamin-A) AND (variation OR mutation) AND (cardiovascular OR collagenopathy OR Ehlers-Danlos OR aortic)) allowed the identification of 97 and 59 bibliographic References, respectively. All article types, in English or French language only,

were considered. Two reviewers screened titles and abstracts after removing duplicates. Among them, 74 and 37 respectively were excluded as they were irrelevant. Articles in which the full text wasn't available were excluded and patients reported more than once were described once. General reviews, and articles focusing on animal models, *in vitro* analyses, irrelevant phenotypes, other genes than *FLNA*, were considered irrelevant and excluded. We excluded references related to Mitral-Valve Dysplasia X-linked, where pathogenic variations do not lead to complete LoF unlike the PVNH1 phenotype. X-linked congenital intestinal pseudo-obstruction, isolated macrothrombocytopenia and phenotype linked to gain-of-function variants in *FLNA* were also excluded. Twenty-six references (1,3-6, 9-11,14-31) were retained and citation scanning of the corresponding articles allowed to add seven references to this selection(32-37) . The final list of 33 articles was composed of 12 case-reports, one phenotype update and 20 case series and involved a total of 245 index-cases with *FLNA* variants.

Step 2 targeted the precise identification of patients with CTD and/or CV anomalies. We chose to include only index-cases with *FLNA* variants associated with at least one CV or CTD feature. Duplicates were removed. We excluded a total of 184 patients including 76 with no CV or CTD features and 108 from two large cohorts where individual phenotypes were unavailable[14-15]. We finally identified 61 out of the 245 index-cases as having unambiguous CV and/or CTD features.

In step 3, all molecular diagnosis results from the selected patients were reviewed and reclassified using HGVS nomenclature, ACMG classification in Human Genome build 19. The same *FLNA* NM_001110556 isoform was used as our referral isoform. Patients with variants reclassified as benign were excluded. Patients with variants reclassified as Variants of Unknown Significance (VUS) were kept.

In step 4, clinical evaluation of the selected cases was done using the standardized questionnaire (supplementary method 1) used for cohorts 1 and 2 characterization.

Supplemental method 3. Questionnaire used to gather clinical information on cardiovascular and CTD features

*Fill all boxes and answer by **Y** = Yes, N = No, X = Unknown*

Questionnaire for genetic study orientation

ARTERIAL DISSECTION AND ANEURYSM AND/OR EHLERS-DANLOS SYNDROME

Doctor ……………………………………………………

Department ………………………………………………

Last name …………………………………………………

Maiden name ……………………………………………

First name…………………………………………………

Sex (M or F) |__|

Date of birth |__|__|__|__|__|__|__|__|

Consanguinity (Y/N) |__|

Other affected family members (Y/N) |__|

(Please enclose a pedigree)

Known mutations (Y/N) |__|

Mutation:……………………………………………

Date of clinical manifestations |__|__|__|__|

Date of diagnosis |__|__|__|__|

1 – GENERAL

Weight (kg) ……………………

Size (cm)

Arterial hypertension (Y/N) |__|

Smoking (Y/N) |__|

Diabetes (Y/N) |__|

SED and/or TAAD family history (Y/N) |__|

Comments: ……………………………………………

2 – CUTANEOUS AND WALL FRAGILITY

Thin translucent skin (Y/N) |__|

Hyperelasticity of the skin (Y/N) |__|

Spontaneous / Extensive bruising (Y/N) |__|

Wall hernias (Y/N) |__|

Localization: ……………………

Abnormal scars (Y/N) |__|

Characteristic facial appearance (Y/N) |__|

Precise:

……………………………………………………………

3 – ARTICULAR SYMPTOMS

Joints hyperlaxity Y/N) |__|

Beighton score: ……………………………………

Tendon or muscles rupture (Y/N) |__|

Scoliosis (Y/N) |__|

Pectus excavatum / Carinatum (Y/N) |__|

4 –CARDIOVASCULAR COMPLICATIONS

Age of the first arterial complication |__|__|

Early onset varicose veins (Y/N) |__|

Varicose veins surgery complications (Y/N) |__|

Aortic aneurysm / dissection (Y/N) |__|

Valsalva diameter (mm)……………………………

Zscore ……………………

Aortic surgery (date/nature/indication)……………………

Other Arterial aneurysm / dissection (Y/N) |__|

Localization(s):

Iliac artery (Y/N) |__|

Renal artery (Y/N) |__|

Splenic artery (Y/N) |__|

Internal carotid artery (Y/N) |__|

Femoral artery (Y/N) |__|

Pulmonary Artery(Y/N) |__|

Arterial tortuosity (Y/N) |__|

Bicuspid aortic Valve |__|

Mitral Valve anomaly |__|

Tricuspid Valve anomaly |__|

Aortic valve anomaly |__|

Other Valvular anomalies (Y/N) |__|

Type: ……………….

Localization(s): ………………………

Patent ductus arteriosus (Y/N) |__|

Ventricular septal defect (Y/N) |__|

Other:……………………………………………………

5 – VISCERAL SYMPTOMS

Pneumothorax (Y/N) |__|

Pulmonary hypertension(Y/N) |__|

Emphysema (Y/N) |__|

Chronic abdominal pain (Y/N) |__|

Digestive fragility or rupture (Y/N) |__|

Number |__|__|

Spontaneous (Y/N) |__|

Sigmoid colon (Y/N) |__|

Comments:…………………………………………………

……………………………………………

6 – NEUROLOGICAL

Learning disorder (Y/N) |__|

Epilepsy (Y/N) |__|

Cerebral MRI (Y/N) |__|

PNH (Y/N) |__|

Mega cisterna magna (Y/N) |__|

Others………………………………

7 – OTHER

Macrothrombocytopenia (Y/N) |__|

Vascular Ehlers-Danlos syndrome - Centre National de Référence des Maladies Vasculaires Rares, HEGP, Paris.

Supplemental method 4. Detailed genetic analysis of Cohort 1 patients

1. Written consents

Written informed consent for genetic study were obtained for all patients and diagnostic genetic testing was performed in accordance with the French legislation on genetic diagnostic tests (French bioethics law no. 2004-800).

2. DNA extraction

The Qiamp DNA Blood Midi kit (Qiagen, Hilden, Germany) was used according to the manufacturer's instructions to extract genomic DNA from leucocyte pellets. All DNA samples were adjusted to a concentration of 12.5 ng/µL.

3. Molecular analysis

From 2014 to 2017, diagnostic CTD gene sequencing was performed by NGS using the HaloPlex Custom Kits (Agilent®). The panel includes all coding and flanking intronic regions of 35 CTD genes (listed below).

**Gene**	***Hg37 (transcrit)***	**Gene**	***Hg37 (transcrit)***
ACTA2	*NM_001613*	**FLNA**	**NM_001110556.1**
ADAMTS2	NM_014244.4	MAT2A	NM_005911.4
B3GALT6	NM_080605.3	MATR3	NM_199189.2
B4GALT7	NM_007255	MFAP5	NM_003480.2
CHST14	NM_130468.3	MYH11	NM_022844
COL1A1	NM_000088.3	MYLK	NM_053025.3
COL1A2	NM_000089.3	NOTCH1	NM_017617.3
COL3A1	NM_000090.3	PLOD1	NM_000302.3
COL4A1	NM_001845.4	SKI	NM_003036.3
COL5A1	NM_000093.4	SKIL	NM_005414.3
COL5A2	NM_000393.3	SLC2A10	NM_030777.3
COL6A1	NM_001848.2	SMAD3	NM_005902.3
DSE	NM_001080976.1	TGFB2	NM_001135599.2
EFEMP2	NM_016938.4	TGFB3	NM_003239.3
ELN	NM_000501.3	TGFBR1	NM_004612.2
FBLN5	NM_006329.3	TGFBR2	NM_001024847.2

FBN1	NM_000138.4	TNXB	NM_019105.6
FKBP14	NM_017946.3		

From 2018, diagnostic CTD gene sequencing was performed by NGS using capture array (NimbleGen SeqCap®, Roche, France). This second version of CTD panel includes all coding and flanking intronic regions of 45 CTD genes (listed below).

**Gene**	***Hg37 (transcrit)***	**Gene**	***Hg37 (transcrit)***
ACTA2	NM_001141945.2	LTBP2	NM_000428.2
ADAMTS2	NM_014244.4	LTBP3	NM_001130144.2
ADAMTSL4	NM_001288608.1	MAT2A	NM_005911.5
BGN	NM_001711.5	MFAP5	NM_003480.3
C1R	NM_001733,4	MYH11	NM_001040114.1
C1S	NM_001734.4	MYLK	NM_053025.3
COL1A1	NM_000088.3	NOTCH1	NM_017617.3
COL1A2	NM_000089.3	PLOD1	NM_000302.3
COL3A1	NM_000090.3	PRKG1	NM_006258.3
COL4A1	NM_001845.4	PRKG1	NM_001098512.2
COL4A2	NM_001846.2	ROBO4	NM_019055.5
COL5A1	NM_000093.4	SKI	NM_003036.3
COL5A2	NM_000393.4	SLC2A10	NM_030777.3
EFEMP2	NM_016938.4	SMAD2	NM_001003652.3
ELN	NM_000501.3	SMAD3	NM_005902.3
FBLN5	NM_006329.3	SMAD4	NM_005359.5
FBN1	NM_000138.4	SMAD6	NM_005585.4
FBN2	NM_001999.3	TGFB2	NM_001135599.3
FKBP14	NM_017946.3	TGFB3	NM_003239.4
**FLNA**	**NM_001110556.1**	TGFBR1	NM_004612.2
FOXE3	NM_012186.2	TGFBR2	NM_001024847.2
HCN4	NM_005477.2	TNXB	NM_019105.6
LOX	NM_002317.6		

4. Variants interpretation and classification:

All variants were interpreted with *in silico* prediction tools using Alamut® Visual version 2.10 (Interactive Biosoftware, Rouen, France). Only variants of interest were mentioned. The reference FLNA transcript used for the analysis was NM_001110556.1. All FLNA variant were classified using HGVS and ACMG classification in Human Genome build 19, and their

frequencies were determined using GnomAD database (V2.1.1). For missenses variants in the CH1 domain, we used PM1 criteria, the databases consulted having a high threshold of pathogenic variants in this domain: In GnomAD database, only 0.8% (n=7/867) of reported missenses variants was in the CH1 domain. Moreover, in the ClinVar database, 3 non-VUS missenses variants was in the CH1 domain (3 pathogenic or likely pathogenic and 0 benign) and in the Actin-binding region (Nter + CH1+CH2),13 non-VUS missense variants was reported : 1 benign and 12 likely or pathogenic. Same proportion of pathogenic/benign missenses variant in this region was reported in the LOVD database (9 likely and pathogenic missenses variant / 109.

Supplemental table 1: Neurological, Cardiovascular and Connective Tissues Disorder features compared between male and female patients from the literature.

		Females (n=39) % (n)	Males (n=20) % (n)	p-value
**Neurological findings**	Seizures	46% (18/39)	47% (8/17)	0.873
	Mega cisterna magna	30% (9/30)	33% (5/15)	0.051
**Cardiovascular findings**	Aortic dilation/aneurysm	57% (20/35)	39% (7/18)	0.208
	Pulmonary artery dilation/PAH	38% (13/34)	38% (5/13)	0.953
	Aortic valve dysfunction	23% (8/35)	13% (2/15)	0.702
	Mitral valve anomalies	31% (9/29	59% (10/17)	0.053
	Tricuspid valve anomalies	7% (2/27)	53% (9/17)	**0.001**
	Bicuspid aortic valve	4% (1/25)	0% (0/15)	1,00
	PDA	40% (14/35)	44% (7/16)	0.800
	VSD ASD	27% (9/33)	27% (4/15)	1,00
	Early-onset varicose veins	100% (1/1)	0% (0/0)	1,00
	Arterial tortuosity	50% (2/4)	100% (1/1)	1,00
	Arterial aneurysm/dissection	100% (3/3)	0% (0/1)	1,00
**Connective Tissues Disorder features**	Joints hyperlaxity (JHL)	67% (18/27)	85% (11/13)	0.445
	Skin hyperelasticity (SHE)	35% (8/23)	60% (6/10)	0.319
	Spontaneous or easily bruising	36% (4/11)	0% (0/6)	0.25
	Cutaneous fragility (scar)	80% (8/10)	33% (2/6)	0.118
	Wall hernia	50% (4/8)	56% (5/9)	0.580
	Emphysema	64% (16/25)	38% (3/8)	0.219
	PNO	14% (1/7)	33% (1/3)	1,00

* Other CV complications included Ventricular hypertrophy or hypoplasia, patent foramen ovale, severe hypoplasia of the transverse aortic arch

Supplemental Table 2 : Statistical analysis of clinical features distribution in the French cohorts 1 and 2 and in the literature patients.

		**Cohort 1 vs [Cohort 2+Literature patients]**	**Cohort 2 vs Literature patients**
	**Age**	0.00046	0,23
**Neurological findings**	Seizures	0.85	0.07
	Mega cisterna magna	0.97	0.62
**Cardiovascular findings**	Aortic dilation/aneurysm	0.06	0.29
	Pulmonary artery dilation/PAH	0.66	**0.03**
	Aortic valve dysfunction	0.07	**0.02**
	Mitral valve anomalies	0.11	**0.03**
	Tricuspid valve anomalies (Prolapse, dysplasia)	0.53	0.14
	Bicuspid aortic valve	0.99	**0.01**
	PDA	0.99	0.40
	VSD/ASD	1.00	0.99
	Early-onset varicose veins	0.70	0.99
	Arterial tortuosity	0.33	1.00
	Arterial aneurysm/dissection	0.26	0.18
**Connective Tissue Disorder Features**	Joints hyperlaxity (JHL)	0.84	0.16
	Skin hyperelasticity (SHE)	0.79	0.55
	Spontaneous or easily bruising	0.42	0.98
	Cutaneous fragility (scar)	0.93	**0.01**
	Wall hernia	0.31	0.06
	Emphysema	0.92	**0.01**
	PNO	1.00	1.00
	Scoliosis	0.05	0.30
	Pectus carinatum/excavatum	1.00	1.00
	GI features	0.13	**0.003**

Supplemental Table 3: Type of *FLNA* variants in the three groups of patients.

Type of variant	Cohort 1	Cohort 2	Literature Patients	Total
**Frameshift**	4	11	19	34
**Nonsense**	3	2	10	15
**Missense**	0	3	17	20
**canonical splice-site**	1	2	7	10
**large deletion**	2	3	4	9
**intronic**	0	1	1	2
**synonymous**	0	1	1	2
**inframe indel**	0	0	1	1

Supplemental Table 4: Distribution of the pathogenic and probably pathogenic missense variants in FLNA protein domains from the three groups of patients.

Protein domain	Size of the domain (AA)	Number of variants (number of affected positions)	p-value
**Nter**	42	2 (1)	0,226
**CH1**	107	12 (10)	**2.289E-8**
**CH2**	104	0	1
**ROD1**	1465	2 (1)	**0.0156**
**Hinge 1**	38	1	0.208
**ROD2**	738	1	0.220
**Cter**	131	1	0.539

Supplemental table 5 : Table of abbreviations

Abbreviation	Definition
ASD	Atrial septal defect
CH1 and CH2	Calponin homology domains
CTD	Connective tissue disorders
CV	Cardiovascular
EDS	Ehlers-Danlos syndrome
JHL	Joint hyperlaxity
LoF	Loss-of-Function
Nter	N terminal domain
PAH	Pulmonary arterial hypertension
PDA	Patent ductus arteriosus
PNH	Periventricular nodular heterotopia
PNO	Pneumothorax
PVNH1	Periventricular nodular heterotopia type 1
SHE	Skin hyperelasticity
TAAA	Thoracic ascending aortic aneurysm
VSD	Ventricular septal defect
VUS	Variant of unknown significance
